# Antioxidant and Antimicrobial Activities of the Essential Oil of *Achillea millefolium* L. Grown in France

**DOI:** 10.3390/medicines4020030

**Published:** 2017-05-19

**Authors:** Chaker El-Kalamouni, Petras Rimantas Venskutonis, Bachar Zebib, Othmane Merah, Christine Raynaud, Thierry Talou

**Affiliations:** 1 Laboratoire de Chimie Agro-Industrielle, Université Fédérale de Toulouse Midi-Pyrénées, INP-ENSIACET, Toulouse 31030, France; Othmane.Merah@ensiacet.fr (O.M.); Christine.raynaud@ensiacet.fr (C.R.); Thierry.Talou@ensiacet.fr (T.T.); 2 Unité Mixte 134 Processus Infectieux en Milieu Insulaire Tropical, Université de la Réunion, INSERM U1187, CNRS UMR 9192, IRD UMR 249. Plateforme Technologique CYROI, Sainte Clotilde 97490, France; 3 Department of Food Technology, Kaunas University of Technology, Radvilènu pl. 19, LT-50254 Kaunas, Lithuania; rimas.venskutonis@ktu.lt; 4 Laboratoires Agronutrition SAS, 3 allée de l’Orchidée Carbone 31390, France; b.zebib@agro-nutrition.fr

**Keywords:** *Achillea millefolium* L., essential oil, antioxidant, antimicrobial activity

## Abstract

**Background:** This study aimed to examine the composition of essential oil (EO) of *A. millefolium* aerial parts wild plant grown in France and evaluate its antioxidant, antibacterial, and antifungal activities. **Methods:** GC-MS was used to identify the chemical composition of EO. Antioxidant activity (AA) of EO was evaluated by Oxipres method. Antimicrobial activity of EO was evaluated by Agar-well diffusion and a broth microdilution assay. **Results:** Forty-three volatile compounds were identified. Major compounds were camphor (12.8%), germacrene-D (12%), (E)-nerolidol (7.3%), sabinene (6.7%), (E)-p-mentha-2,8-dien-1-ol (4.5%), and 1,8-cineole (4%). EO shows strong AA against Sunflower oil oxidation. Additionally, an inhibitory effect against microbial organisms (bacteria and fungi) was found. **Conclusion:** The EO composition of *A. millefolium* chemotype located in France was studied. The EO of the *A. millefolium* wild plant grown in France is quite an effective antioxidant in sunflower oil oxidation; it also possesses inhibitory effects against famous bacteria and fungi.

## 1. Introduction

The global interest in food preservation has been recently greatly increased due to high economic costs of deterioration and poisoning of food products through lipid oxidation as well as food pathogens. Currently, there is a growing interest in prolonging shelf-life and the safety of food using natural antioxidant and antimicrobial compounds. This aspect assumes particular relevance due to an increased resistance of some bacterial and fungi strains to the most common antibiotics and antimicrobial synthetic agents [1]. Essential oils or some of their components have been largely used in perfumes, in sanitary products, in dentistry, in agriculture, as food preservers and additives, and as naturals remedies [2].

Mostly found in Europe, Asia, and temperate regions including North America, the *Achillea* genus, belonging to Asteraceae family, is represented by about 85 species [3]. *A. millefolium* (common yarrow) has been used in many applications such as medicine, veterinary science, and cosmetics [4]. The flowering herbs were reported as possessing tonic, antispasmodic, vulnerary, and diaphoretic activities, among others, and therefore is recommended for colds, flatulence, hysteria, and rheumatism treatments [5]. The chemical composition of *A. millefolium* oils from Québec [6], India [7], Turkey [3], Iran [8], and European countries (Macedonia, Italy, Lithuania, Hungary, Greece, Moldavia, Latvia, Germany Estonia, Belgium, France, Russia, Armenia, Spain, and Bulgaria) [9,10,11,12,13,14] has been reported in previous studies. However, the reports on antioxidant and antimicrobial properties of *A. millefolium* EO are rather scarce. Considering remarkable chemical polymorphism, which is characteristic of many essential oil-bearing plants, it is of interest to determine the chemical composition and biological activities of plant varieties growing in different regions. 

Therefore, the aim of this study was to investigate the antioxidant, the antibacterial, and the antifungal activities of EO of *A. millefolium* aerial parts of French origin. Additionally, this study aims to inspect the chemical profile of *A. millefolium* EO grown in France and to compare this profile to previous studies where the EO of *A. millefolium* was obtained from other countries. 

## 2. Materials and Methods

### 2.1. Plant Material

The aerial parts of wild *Achillea millefolium* L. plant were picked in May 2008 at the bloom stage from Toulouse (43°36′01′′ N, 1°25′58′′ E), France. The voucher specimens were deposited in the Herbarium of the Pharmacognosy Laboratory, Faculty of the Pharmaceutical Sciences, Université de Toulouse III, N° (AST. 251 (2008) (I.F.)).

### 2.2. EO Extraction

Five hundred grams (500 g) of fresh areal plant parts at flowering stage were blended with 4 L of distilled water and submitted to water-distillation in a modified Clevenger-type apparatus for 3 h. After distillation, the collected oil was isolated from the water and dried over sodium sulfate (water free). Six replicate distillations were performed and the EO was stowed at 4 °C before analysis.

### 2.3. Gas Chromatography Coupled with Mass Spectrometry Analysis (GC-MS)

The EO was solvated in *n*-pentane (Sigma-Aldrich, St Quentin Fallavier, France) (10% *v*/*v*) and construed on a Clarus 500 (Perkin Elmer Instruments, Shelton, USA) gas chromatograph outfit with a fused-silica capillary column HP-5MS (5% diphenyl 95% dimethyl polydimethylsiloxane, 30 m length, 0.25 mm id, 0.25 µm film thickness (J&W scientific, Folsom, CA). Flame ionization detector (FID) (Perkin Elmer Instruments, Shelton, CT, USA) was used. The importer gas was helium (1.3 mL/min flow). The injection volume was 0.5 µL. Injector temperature was 250 °C and under split mode at a ratio of 1:5. The temperature of FID was 250 °C. The furnace temperature was programmed at 60 °C for 5 min, grated to 250 °C at 5 °C/min and maintained at 250 °C for 5 min. The identification of compounds was built on retention indices (RI) qualified to C5-C18 n-alkanes (Sigma-Aldrich, St Quentin Fallavier, France) and available literature data [15,16] and on computer matching of obtained fragments from mass spectrum with those present in NIST 2008 and WILEY 275L MS libraries. Results are the mean from at least three independent experiments performed in six replicates.

### 2.4. Evaluation of AA of EO by the Oxipres Method

The preparation of samples is obtained by mixing sunflower oil with 0.05% or 0.1% or 0.2% amounts of EO. In the Oxipres apparatus (Mikrolab, Aarhus, Denmark), 5 g of sunflower oil were placed inside a reactor tube heated at 110 °C under an oxygen pressure of 5 bar. The variations in pressure occurred were recorded. The control sample not contain additive. After the addition of each dose of EO, all variations of induction period (*IP*) of sunflower oil were collected with time (h). 3,5-di-tert-4-butylhydroxytoluene (BHT) (Sigma-Aldrich, St. Quentin Fallavier, France) was used as a positive control. The AA of EO and the protection factor (*PF*) variations of sunflower oil were determined by Equations (1) and (2):

(1) $$PF=\frac{{IP}_{x}}{{IP}_{k}}$$

(2) $$AA=\frac{{IP}_{x}-{IP}_{k}}{{IP}_{BHT}-{IP}_{k}}$$

where *IP_X_* is the induction period of the sample with additive, *IP_K_* is the induction period of the sample without additive, *IP_BHT_* is the induction period of the sample with synthetic antioxidant BHT.

### 2.5. Assessment of Antibacterial Effect of EO

Two types of bacteria were tested; (1) Gram-positive—*Bacillus subtilis* ATCC6633, *Bacillus cereus* ATCC10876, *Staphylococcus aureus* ATCC25923, and *Staphylococcus epidermidis* ATCC12228—and (2) Gram-negative pathogens *Salmonella typhimurium* ATCC14028, *Salmonella typhimurium* DS88, *Salmonella enteritidis* ATCC13076, *Salmonella agona* ATCC43889, and *Escherichia coli* ATCC25922 were kindly donated by Dr. A. Šarkinas (Food Institute of Kaunas University of Technology, Kaunas, Lithuania).

#### 2.5.1. Agar Well Diffusion Method

Antibacterial assay was carried using agar well diffusion. The bacteria were cultured for 18 h at 37 °C on the slant agar (Oxoid, CM325). Adjustment of washed cell bacteria was carried out according to McFarland No. 0.5 standard [17]. Into 90 mm diameter Petri plates, 10 mL of each bacteria culture were pipetted. To fill the wells (6 mm in diameter), agar was pressed with 10 µL of ethanolic solutions contained 5 µg/mL of EO. Ethanol was used to dissolve the oil for negative controls preparation. Susceptibility test disc Ceftazidime/Clavulanic Acid, CAZ-CLA, 30/10 µg (BBL, CAZ/CLA, 231753) was used as a positive control. Incubation time of plates was 24 h at 37 °C. Antibacterial effect was assessed by the diameter of clear zones developed around wells. Results are the mean (±SEM) from at least three independent experiments performed in triplicates.

#### 2.5.2. Determination of Minimum Inhibitory Concentrations (MICs)

MIC was determined on the base of a microdilution broth susceptibility test [18]. All tests were made in Mueller Hinton Broth (MHB; BBL) (Microbiology Systems, Inc., Cockeysville, MD, USA) supplemented with Tween 80 detergent (Fisher, Illkirch, France) to achieve a final concentration of 0.5% (*v*/*v*). The culture of bacteria was made overnight at 37 °C in Mueller Hinton Agar, MHA (Sigma-Aldrich, St. Quentin Fallavier, France). To obtain a final density of 5 × 10^5^ cfu/mL, tests were hanging in MHB. To obtain a final concentration values reaching from 0-1000 µg/mL of EO in MHB, dilutions were prepared in a 96-well microplates (Sarstedt, Nümbrecht, Germany). Growth control was (MHB + Tween 80) and sterility control was (MHB + Tween 80 + sample). The microplates were smoldered at 37 °C during 24 h. A microplate reader (Multiskan Ascent, Thermo Fisher Scientific, Loughborough, UK) was used to the bacterial growth determination. The reading absorbance was 620 nm. The MICs represent the concentrations of EO causing at least 50% growth inhibition of the bacterial strains. Results are the mean (±SEM) from at least three independent experiments performed in triplicates.

### 2.6. Assessment of Antifungal Activity of EO

The fungi samples were obtained from the Leibniz Institute (DSMZ, Braunschweig, Germany). The fungal species tested in this study were *Botrytis cinerea* DSM-5144, *Rhizopus stolonifer* DSM-2194, *Verticillium dahliae* DSM-11938, *Aspergillus niger* DSM-1957, and *Colletotrichum gloeosporioides* DSM-62146. The culture of fungal species was made on Potato-Dextrose-Agar (PDA) slants purchased from VWR (Strasbourg, France). The storage temperature was 4 °C. After 10 days of culture, the fungi spore were obtained, then blended with sterile distilled water to get a spore suspension of 1 × 10^8^ spore/mL. The concentrations of EO solutions were reached from 0.1 to 3 mg/mL. The disc diffusion method [16] was adopted to evaluate the antifungal activity. Five microliters (5 µL) of EO was used. Inhibition percent was obtained by Equation (3):

(3) $$Inhibition\;\left(\%\right)=\frac{1-radial\;growth\;of\;treatment\;}{radial\;growth\;of\;control\;}\times 100.$$

MIC value was obtained from the equation of the line (% inhibition against concentration). The lower MIC value shows better antifungal activity. All antifungal results are the mean (±SEM) from at least three independent experiments performed in triplicates.

## 3. Results and Discussion

### 3.1. Composition of EO

The yellow greenish EO isolated from French *A. millefolium* aerial parts possessed a strong characteristic odor. The EO yield was 0.07% (Fresh weight), which is 5 t higher than reported for Indian origin (0.014%) [5], 3 t lower than in Canadian samples (0.21%) [6].

Forty-three compounds were identified in *A. millefolium* EO amounting 96.3% of the total EO (Table 1). Oxygenated monoterpenes constituted the major part of the EO (40.7%), with camphor (12.8%), *trans*-chrysantenyl acetate (6.6%), terpinen-4-ol (4.70%), (*E*)-*p*-mentha-2,8-dien-1-ol (4.5%), and 1,8-cineole (4.0%) being its main components. Other quantitatively important fractions were hydrocarbon sesquiterpenes (17%) and oxygen-containing sesquiterpenes (19.5%), germacrene-D (12.0%) and (*E*)-nerolidol (7.3%) being the main components. The monoterpene part corresponded only to 15%, sabinene (6.7%) and β-pinene (3.4%) being its main constituents.

It may be observed that the main components of *A. millefolium* analyzed in our study to some extent were comparable to those previously reported [7,8,9,10,11,12]. However, the content of such components as hexane-2,3-dione, hex-3-en-1-ol, methyl hexanoate, (*E*)-*p*-mentha-2,8-dien-1-ol, (*Z*)-*p*-menthan-2-one, thymol acetate, (*E*)-methyl isoeugenol, β-cedrene epoxide, and patchouli alcohol in the oil of *A. millefolium* analyzed in our study was considerably higher than in the previously studied yarrow oils. Camphor (up to 20%), α and β-thujones (up to 26.8%), 1,8-cineole (up to 20.3%), artemisia ketone (up to 10.1%), and chamazulene (up to 0.8%) were reported in *A. millefolium* oil from France [12], but the content of these compounds in our study was rather different; it contained 12.8% camphor, 4.5% (*E*)-*p*-mentha-2,8-dien-1-ol, 4.7% terpinen-4-ol, 4.0% 1,8-cineole, and 1.8% borneol, whereas artemisia ketone, thujones, and chamazulene were not detected. 

A completely different *A. millefolium* chemotype was reported in the Saguenay region around Quebec in Canada where the β-thujone was the main compound (13.8%) [6]. The sesquiterpene alcohols spathulenol, (*Z*)-nerolidol, α-bisabolol, and (*E,E*)-farnesol were dominant constituents in the *A. millefolium* oil from Iran [8]. These results confirm that the chemical polymorphism is a characteristic feature of yarrow. The variations in the chemical profile of the *A. millefolium* oil as a consequence of ontogenesis and organic differentiation were recently noted [19]. The influence of ecological factors (climate, altitude, association, nutrients, etc.) seems to be an important factor; in addition to the like chemotype, variations in EO chemical composition could exist.

### 3.2. AA of EO Against Sunflower Oil Oxidation

Plant oils containing sensitive to oxidative degradation unsaturated fatty acids have been widely used as substrates for testing AA. The ability of *A. millefolium* EO to protect the sunflower oil was evaluated by comparing with synthetic antioxidant BHT in Oxipres apparatus. The effect of additives was expressed as a protection factor (PF) indicating the increase in sunflower oil stability (Table 2). The addition of 0.2% of *A. millefolium* EO increased the stability of sunflower oil about 1.5 t (the IP of sunflower oil without additives was 2.9 h). *A. millefolium* oil prolonged the IP of sunflower oil oxidation depending on the concentration; the highest concentration of additive (0.2%) was more effective than BHT applied at 0.02% (Table 2). AA of other EO plant species in sunflower oil was previously evaluated by the Oxipres method; for instance, *Calamintha grandiflora* applied at 0.2% demonstrated PF of 1.63 [20]. In conclusion, *A. millefolium* EO stabilized the oxidation of sunflower oil. This stability of oil rose with the added amounts of EO.

### 3.3. Antibacterial Activity of EO

Antibacterial effect of EO was evaluated in vitro against nine pathogenic bacteria species (Table 3). Gram-positive bacteria were more delicate to the EO than Gram-negative bacteria. *B. cereus* was the most susceptible bacteria to *A. millefolium* oil at the applied concentrations (5 and 10 µg/mL). The smallest inhibition zones were obtained in *S. typhimurium* and *S. agona* cultures while *St. epidermidis, S. enteritidis*, and *E. coli* were resistant against all concentrations of *A. millefolium* oil. MIC for *B. cereus* and *St. aureus* were 100 and 120 µg/mL, respectively; while the MIC for *B. subtilis* was 310 µg/mL. Among the Gram-negative tested bacteria, a remarkable antibacterial effect was observed only for *S. typhimurium* and *S. agona* with their respective MIC values 2000 and 1000 µg/mL. The effect of *A. millefolium* oil from Turkey [3] was also tested against *St. aureus* and *B. cereus*; it was found that the MIC was 72 mg/mL, which is remarkably higher than the MIC of *A. millefolium* oil measured in our study. As was shown, the composition of *A. millefolium* oils from various locations may be rather different. The properties and the content of individual constituents may have an important power on the antibacterial effect of oil against different bacteria species. For instance, sabinene, 1,8-cineole, and camphor, which were abundant in *A. millefolium* oil studied in this work, are well-known natural chemicals with antibacterial potentials [21]. The EO also studied in our work also contained 12% of germacrene-D and 7.3% of (*E*)-nerolidol, which were absent in the Turkish oil. The antibacterial activities of germacrene-D and (*E*)-nerolidol were previously reported as well [22].

### 3.4. Antifungal Activity of EO

EO of *A. millefolium* showed remarkable antifungal activity against all plant fungi tested (Table 4) at 5 µL (1000 ppm/disc). It exhibited an inhibitory result on the development of *R. stolonifer* (65.7%) *V. dahliae* (56.3%), *C. gloeosporioides* (60.9%), *Botrytis cinerea* (50.8%), and *Aspergillus niger* (40.7%). The inhibitory effects might be related to the presence of mono- (55.7%) and sesquiterpenes (36.5%) in the EO [23]. 

The MICs defined as the deepest concentrations of the EO that allows for full progress inhibition of *R. stolonifer*, *V. dahliae*, *C. gloeosporioides*, *B. cinerea*, and *A. niger* were 1.6, 3.1, 3.4, 3.6, and 4.7 mg/mL, respectively (Table 5). *R. stolonifer* was discovered to be the greatest sensitive fungal pathogens towards the *A. millefolium* EO.

## 4. Conclusions

In conclusion, the EO of *A. millefolium*, growing wild in the Midi-Pyrenees region, possesses quite a unique chemical composition as compared with the oil composition of the same species reported in previously published studies. α-Pinene, sabinene, camphor, *trans*-chrysanthenyl acetate, cyclocitral isomers, and germacrene D were the main components indicating an individual chemotype of the studied plant sample. The EO of *A. millefolium* was quite an effective antioxidant preserving oxidation of sunflower oil; it also possessed inhibitory effects against tested bacteria and fungi. The EO of *A. millefolium* growing wild in the Toulouse region show a varied interval of activities against plant fungi. In our study, it has become clear that the EO of *A. millefolium* possesses an important ability to intensely inhibit the development of *B. cinerea* with other plant fungi tested in this work. These results provide preliminary support for the potential uses of *A. millefolium* essential oil for the prolongation of shelf-life and the safety of food preparations possessing specific odor properties, as well as antioxidant and antimicrobial activities.

## Figures and Tables

**Table 1:** Chemical composition of EO of wild *Achillea millefolium* L. (aerial parts) from Toulouse region, France.

No.	Compounds	RI ^a^	RI ^b^	(%) ^c^	RSD ^d^
1	Hexane-2,3-dione	800	790	0.1	6.39
2	(E)-Hex-3-en-1-ol	849	854	0.2	7.15
3	Methyl hexanoate	926	927	0.2	5.68
4	α-Pinene	935	939	1.7	6.93
5	Camphene	952	954	1.6	8.35
6	Sabinene	974	975	6.7	7.31
7	β-Pinene	981	979	3.4	9.66
8	α-Terpinene	1018	1017	0.7	6.43
9	*p*-Cymene	1025	1025	0.8	8.20
10	1,8-Cineole	1034	1031	4.0	8.89
11	γ-Terpinene	1058	1060	1.7	7.93
12	Terpinolene	1086	1089	0.4	6.39
13	Linalool	1099	1097	1.8	7.15
14	Nonanal	1103	1101	0.6	5.68
15	(E)-*p*-Mentha-2,8-dien-1-ol	1119	1123	4.5	6.93
16	(Z)-*p*-Mentha-2,8-dien-1-ol	1138	1138	0.3	8.35
17	*trans*-Pinocarveol	1144	1139	0.8	5.30
18	Camphor	1151	1146	12.8	4.85
19	Borneol	1163	1169	1.8	4.83
20	Terpinen-4-ol	1183	1177	4.7	4.10
21	(Z)-*p*-Menthan-2-one	1198	1196	1.5	4.56
22	trans-Chrysanthenyl acetate	1228	1238	6.6	1.76
23	Bornyl acetate	1285	1289	1.2	5.60
24	Thymol acetate	1355	1352	0.7	6.27
25	α-Copaene	1377	1377	0.5	5.30
26	(E)-Caryophyllene	1415	1419	1.7	4.85
27	*cis*-β-Farnesene	1447	1443	0.8	4.83
28	Germacrene-D	1483	1485	12.0	3.97
29	(E)-Methyl isoeugenol	1496	1492	1.0	3.72
30	γ-Cadinene	1513	1514	0.4	3.31
31	δ-Cadinene	1517	1523	1.2	4.17
32	α-Calacorene	1542	1546	0.4	3.60
33	Elemol	1553	1550	1.6	3.95
34	(E)-Nerolidol	1559	1563	7.3	3.36
35	Caryophyllenyl alcohol	1574	1572	0.8	4.00
36	Spathulenol	1584	1578	2.0	3.80
37	Caryophyllene oxide	1596	1583	1.9	4.34
38	Viridiflorol	1610	1593	1.8	4.31
39	β-Cedrene epoxide	1625	1623	0.5	4.62
40	β-Eudesmol	1654	1651	1.1	2.51
41	Cedr-8(15)-en-10-ol	1659	1652	0.2	3.12
42	Patchouli alcohol	1669	1658	0.8	3.90
43	α-Bisabolol	1683	1686	1.5	1.03
**Total**	**compounds**			**96.30**	
	Oxygenated monoterpenes			40.70	
	Oxygenated sesquiterpenes			19.5	
	Hydrocarbon sesquiterpenes			17	
	Monoterpenes			15	
	Other			4.1	

^a^ calculated retention indices; ^b^ literature values of retention indices [15,16]; ^c^ relative% of the compounds gated from FID area percent data; ^d^ RSD: Relative Standard Deviation (*n* = 6).

**Table 2:** AA of *Achillea millefolium* L. essential oil evaluated in sunflower oxidation test (Oxipres method).

Additive	Concentration %	Protection Factor (PF) ^a^	Antioxidant Activity (AA) ^b^	Induction Period (IP) ^c^	RSD ^d^
Without additive	0.00	1.00	-	2.90	0.30
BHT	0.02	1.47	1.00	4.26	0.50
Essential oil	0.20	1.71	1.51	4.96	0.50
Essential oil	0.10	1.29	0.62	3.74	1.00
Essential oil	0.05	1.17	0.36	3.39	0.40

^a^ PF = IP_(sample with additive)_/IP_(sample without additive)_; ^b^ AA was determined by comparison with the reference (BHT 0.02%); ^c^ IP = time when the pressure start to reduce suddenly; ^d^ RSD: the relative standard deviation (*n* = 3).

**Table 3:** Antibacterial activity of *Achillea millefolium* L. EO against bacterial pathogens.

Microorganism	Gram +/−	Inhibition Zone Diameter (mm) ^a^			Essential Oil
		Essential Oil ^b^		Antibiotic ^c^	MIC ^d^
		10 µg/mL	5 µg/mL	(30/10) µg	µg/mL
*B. subtilis*	G+	11.0 ± 0.2	8.6 ± 0.1	16.8 ± 0.1	310 ± 20
*B. cereus*	G+	13.6 ± 0.2	10.7 ± 0.2	29.0 ± 0.2	100 ± 10
*St. aureus*	G+	12.8 ± 0.1	9.1 ± 0.2	21.0 ± 0.3	120 ± 12
*St. epidermidis*	G+	na ^e^	na	25.8 ± 0.1	na
*S. typhimurium*	G−	9.2 ± 0.2	na	31.2 ± 0.3	2000 ± 40
*S. typhimurium*	G−	na	na	34.0 ± 0.1	na
*S. enteritidis*	G−	na	na	32.2 ± 0.3	na
*S. agona*	G−	10.4 ± 0.4	9.1 ± 0.3	20.8 ± 0.3	1000 ± 40
*E. coli*	G−	na	na	26.4 ± 0.2	na

± was an SD from at least three independent trials; ^a^ Diameter of inhibition area; ^b^ Each well contained 10 µL of ethanolic solution of essential oil (10 and 5 µg/mL); ^c^ Ceftazidime/Clavulanic Acid (30/10 µg/disc) was a positive control; ^d^ MIC: minimum inhibitory concentration (values in µg/mL); ^e^ na: no activity.

**Table 4:** Antifungal activity of *Achillea millefolium* L. EO (5 µL corresponding to 1000 ppm/disc).

Fungal Strain	Mycelial Growth Inhibition ^a^	
	Essential Oil	
	mm	%
*R. stolonifer*	11.5 ± 1.4	65.7 ± 2.2
*V. dahliae*	10.1 ± 1.4	65.3 ± 2.6
*C. gloeosporioides*	12.2 ± 1.4	60.9 ± 2.5
*B. cinerea*	18.4 ± 1.4	50.8 ± 3.8
*A. niger*	24.8 ± 0.8	40.7 ± 0.5

± was an SD from at least three independent trials; ^a^ expressed in mm from radial evolution; % of radial growth inhibition.

**Table 5:** Minimum inhibitory concentration (MIC) of *A. millefolium* L. EO.

Fungal Strain	MIC ^a^
	Oil
*Rhizopus stolonifer* DSM-2194	1.6
*Verticillium dahliae* DSM-11938	3.1
*Colletotrichum gloeosporioides* DSM-62146	3.4
*Botrytis cinerea* DSM-5144	3.6
*Aspergillus niger* DSM-1957	4.7

^a^ MIC, minimum inhibitory concentration (values in mg/mL).
